# Brief Report: Lack of Processing Bias for the Objects Other People Attend to in 3-Year-Olds with Autism

**DOI:** 10.1007/s10803-014-2278-4

**Published:** 2014-10-21

**Authors:** Terje Falck-Ytter, Emilia Thorup, Sven Bölte

**Affiliations:** 1 Department of Women’s and Children’s Health, Pediatric Neuropsychiatry Unit, Center of Neurodevelopmental Disorders at Karolinska Institutet (KIND), Child and Adolescent Psychiatry Research Center, Karolinska Institutet, Gävlegatan 22, 11330 Stockholm, Sweden; 2 Uppsala Child and Babylab, Department of Psychology, Uppsala University, 751 42 Uppsala, Sweden; 3 Division of Child and Adolescent Psychiatry, Stockholm County Council, Stockholm, Sweden

**Keywords:** Communication, Development, Cognition, Neurodevelopmental disorders, Vision

## Abstract

Whether gaze following—a key component of joint attention—is impaired in children with autism spectrum disorder (ASD) is currently debated. Functional gaze following involves saccading towards the attended rather than unattended targets (accuracy) as well as a subsequent processing bias for attended objects. Using non-invasive eye tracking technology, we show that gaze following accuracy is intact in intellectually low-functioning 3-year-olds with ASD. However, analyses of the duration of first fixations at the objects in the scene revealed markedly weaker initial processing bias for attended objects in children with ASD compared to children with typical development and non-autistic children with developmental delays. Limited processing bias for the objects other people attend to may negatively affect learning opportunities in ASD.

Joint attention refers to the triadic sharing of attention between individuals towards an object and is thought to play a fundamental role in socio-communicative development (Mundy et al. 1986). It is a not directly observable psychological construct, and the scientific study of joint attention thus needs operational definitions. Gaze following is thought to be an important aspect of joint attention, and because it is an observable non-verbal behavior, it is a popular measure in research on young children and infants. Typically in such experiments, the gaze of the participant is measured as he or she observes an adult looking at one of several objects, and the tendency to follow the adult’s gaze to that specific object is assessed. Studies using this approach have shown that gaze following develops during the first year of life in typical development (Corkum and Moore 1998; Gredebäck et al. 2008, 2010).

Impairments in joint attention are commonly described in children with autism spectrum disorder (ASD) (Charman 2003; Chawarska et al. 2003). Interestingly, experimental studies suggest that joint attention impairments in ASD do not reflect a fundamental insensitivity to directional information conveyed by the eyes (Chawarska et al. 2003; Senju et al. 2004; for related neuroimaging findings, see Greene et al. 2011). Chawarska et al. (2003) found that while clinical ratings of “response to joint attention” on the autism diagnostic observation schedule (ADOS; Lord et al. 2000) indicated impaired spontaneous gaze following in a sample of children with ASD, automatic gaze cuing did not differ between groups. Leekam et al. (2000) studied the spontaneous tendency to follow the gaze of a model to a specific object and found lower accuracy in autistic compared to developmentally delayed children. However, when splitting the group into high and low IQ samples it became clear that the group difference was mostly evident in the low IQ group. Using a similar design with 8-year-olds, Leekam et al. (1998) found that a majority of the autistic children did spontaneously follow gaze. Using eye tracking, Falck-Ytter et al. (2012) found that gaze following accuracy was related to adaptive communication skills in a sample of preschool children with autism.

In contrast to the joint attention task in ADOS (Lord et al. 2000) and to parental measures of joint attention, experimental studies tend to control for problems attracting the child’s attention in the first place (e.g. by requiring that he or she looked at the experimenter before the gaze/head turn). Thus, the differences between experimental and naturalistic measurements may relate to the extent that they control for this factor.

A recent eye tracking study indicated that the ability to follow gaze is not impaired in infants who later receive an autism diagnosis (Bedford et al. 2012). The authors assessed gaze following in a group of infants at risk for autism (because of having an older sibling with an ASD diagnosis). The children later underwent clinical assessment and were classified as meeting diagnostic criteria for ASD, as having other developmental concerns, or as typically developing. It was found that all groups tended to follow gaze correctly; that is, all groups tended to move their gaze to an attended object rather than an unattended object (i.e., high accuracy in gaze following). It was also found, that at age 13 months, infants with later developing socio-communication problems (including those with an ASD diagnosis) spent less time looking at the attended object than did typically developing infants. This finding led the authors to suggest that the key difficulty in ASD may not be the ability to follow gaze per se but rather to understand the communicative meaning of the gaze shifts of other people.

Previous eye tracking studies of gaze following in ASD have included fixations falling within the areas of interests (AOIs) over several seconds after the gaze cue (Bedford et al. 2012). Such measures are likely to reflect initial object processing *and* processes occurring on longer timescales (e.g. sustained attention). Consequently, a group difference based on accumulated looking time measures does not necessarily reflect a differential processing of the cue per se (the other person’s gaze shift). Therefore, in the current study, rather than focusing on looking time during the whole trial, we chose to measure the duration of the *first* fixation to the attended and unattended objects, respectively. The length of single fixations has previously been related to the degree of information processing of the event or object that is being fixated (Papageorgiou et al. 2014). In reading research, first fixation duration is thought to reflect processing time and the initial lexical activation process (Holmqvist et al. 2011; Rayner 1998). The duration of fixations on words correlates positively with N400 amplitudes (Dambacher and Kliegl 2007), which have also in turn been linked to semantic processing. Research on scene perception suggests that the first fixation duration reflects cognitive processing of the elements in the scene. For example, De Graef et al. (1990) showed that first fixation durations were longer for objects located in unusual contexts (e.g., a motorcycle in a chemistry lab) than for objects in a common context (e.g., a motorcycle at a gas station). The first fixation duration measure was used recently in autism research by Benson et al. (2012). Their participants were presented with pairs of pictures, and in one member of each pair, a detail had been manipulated, making that picture “weird” (e.g., a picture of a highway with a car replaced by a large animal). The authors found that autistic adults differentiated less between the two pictures in terms of first fixation duration than did typically developing controls, which was interpreted as a failure in the ASD group to immediately recognize the “weird” detail.

Against this background, we hypothesized that a first fixation bias favoring the attended object would be weaker in children with ASD than in non-autistic children. To test this hypothesis, we administered a gaze following task to a group of 3-year-olds with ASD, an age-matched control group with typical development (TD group), as well as a group with other developmental delays (DD group) matched on age and developmental level to the ASD group (Study 1). We also assessed gaze following accuracy and latency of gaze shifts, but had no specific hypotheses for these measures (Bedford et al. 2012; Falck-Ytter et al. 2012). In an additional analysis of fixation durations, we compared the performance of the ASD group with that of a group of toddlers with typical development (Study 2).

The study was approved by the local ethics committee in Stockholm and conducted in accordance with the 1964 Declaration of Helsinki. All caregivers gave written consent.

## Study 1

### Methods

#### Participants

A total of 36 children between ages 34 and 60 months (M = 42, SD = 6.7) participated in the study: 13 children with ASD (10 males, 3 females), 9 children with other developmental problems (3 males, 6 females), and 14 typically developing children (11 males, 3 females; all figures refer to final samples after exclusion; see also Table 1). None of the children had any uncorrected hearing or visual impairments or known genetic syndromes. Two of the children with ASD were diagnosed with intellectual disability. Children with ASD were recruited from the Autism Center for Young Children, in Stockholm and had a DSM-IV-TR clinical consensus diagnosis of Autistic Disorder (n = 11) or Pervasive Developmental Disorder, Not Otherwise Specified (n = 2). In all but one child, the diagnosis was corroborated by information from the ADOS (Lord et al. 2000) and/or the Autism Diagnostic Interview-Revised (Lord et al. 1994). The remaining child had autistic disorder, a Social Responsiveness Scale-Preschool version (SRS; Constantino and Gruber 2005) score of 85, and a low IQ score, and thus matched the remaining ASD sample well. Intellectual level (IQ) was not used as an exclusion or inclusion criterion. The typical sample was recruited by notifications and advertisements in the same area as the ASD group.

**Table 1:** Participant characterization

Measure	ASD (n = 13)	DD (n = 9)	TD (n = 14)
Male/female ratio	10/3	3/6	11/3
Age (in months)	43 (36–50)	42 (28–60)	41 (34–55)
SRS T score	75 (58–96)	60 (40–80)	44 (34–59)
MSEL VR	25 (11–46)	22 (11–34)	45 (30–69)
MSEL FM	24 (15–42)	20 (13–31)	43 (34–59)
MSEL RL	19 (9–33)	22 (15–31)	41 (31–53)
MSEL EL	19 (5–39)	20 (4–32)	46 (36–60)

MSEL (Mullen Scales of Early Learning) scores are age equivalents for the scales

Data for age, SRS, and MSEL represent mean (min–max)

*VR* visual reception, *FM* fine motor, *RL* receptive language, *EL* expressive language

The DD group was recruited from habilitation centers in the same geographical area. Professionals at these centers were asked to identify children in their clinics in the age range of interest with no suspected (or confirmed) ASD. These children constitute a heterogeneous group with various conditions, with intellectual disability being common. Our rationale was to recruit a heterogeneous group of young non-autistic children with other developmental problems, expected to match the ASD group on developmental level.

The developmental level of all participating children was determined using the Mullen Scales of Early Learning (MSEL; Mullen 1995). Autism trait severity was assessed using the Social Responsiveness Scale-Preschool version (SRS; Constantino and Gruber 2005). As can be seen, the ASD group scored low on the MSEL; thus, the use of the term ‘low functioning’. To match the ASD group, we excluded two children from the DD group with above average scores on the MSEL. One additional child was excluded due to a lack of congruent gaze shifts (see below).

#### Procedure

Upon arrival at the lab, the parents signed the consent form and handed in questionnaires. After a brief familiarization with the environment and the experimenter, the child and caregiver were taken to the eye tracking lab, and the eye tracking session was initiated. The child was placed at a distance of 60 cm in front of the monitor (17-inch screen), and a 5-point calibration procedure was conducted (repeated if needed to get data for all 5 points). The MSEL was conducted after the eye tracking session. In addition to the present stimuli (see below), the session also included stimuli with biological motion and audiovisual synchrony and attention grabbers (Falck-Ytter et al. 2013). Breaks were embedded flexibly into the sessions (Kylliäinen et al. 2014). The families were given gift vouchers for their participation.

#### Stimuli

**Fig. 1:**
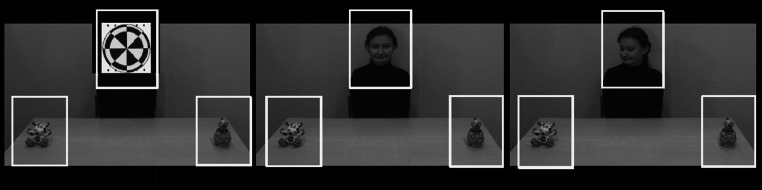
Screen shots of the stimuli material depicting the initial animation covering the model’s face, the model engaging in direct gaze, and the model attending to one of the two objects. Areas of interests (AOIs) are highlighted

The stimuli were eight videos (duration 8 s) of a female model seated behind a table on which two objects were placed (Fig. 1). The video was similar to those used in a study by Senju and Csibra (2008) and consisted of two phases. In the first phase, the model’s face was covered by an animation accompanied by a sound, attracting the child’s attention. This phase lasted 2.30 s. In the second phase, the animation disappeared, revealing the model’s face. The model was looking straight into the camera, smiling at the participant for about 0.20 s before shifting gaze and subsequently turning her head towards one of the two objects (the gaze shift took approximately 0.7 s). The second phase lasted 5.70 s, and the model continued to look at the attended object until the end of the trial. There were two visually identical versions of the video, presented in two separate blocks, the order of which was counterbalanced within group. In the first, a female voice said “Look!” right before the face of the model was revealed. In the second, a distorted version of the same soundtrack was played. The distortion rendered voice and the utterance unrecognizable, but retained the low level properties such as duration and volume. Each version consisted of four trials, counterbalanced for placement of the objects to the left or right side of the table as well as the number of times the two objects were attended. Also, the order of the trials was pseudo-randomized within block. Twenty-one children watched both blocks. However, due to child behavior (e.g. lack of motivation/attention), eight children watched only the block with the voice stimuli, and seven children watched only the block with the distorted voice. Because of limited sample sizes, no statistical comparisons were made between the two versions, and all subsequent data descriptions refer to the combined sets (the average performance across trials, irrespective of soundtrack).

#### Analysis

Gaze data were recorded with a Tobii T120 eye tracker and analyzed using Tobii Studio software (Tobii Technology, Stockholm, Sweden). To allow for large head movements we recorded at 60 Hz rather than 120. To achieve compatibility with earlier studies of the same population (Pierce et al. 2011), a fixation filter (Tobii Fixation Filter) with a velocity threshold of 35 pixels/window and a distance threshold of 35 pixels was applied. Four rectangular AOIs were defined. One covered the animation hiding the face of the model in the first phase, and the remaining three covered the model’s face and the two objects, respectively (in the second phase). The visual angle of the face and animation AOIs subtended approx. 8° by 12°, and the visual angle of the object AOIs subtended approx. 6° by 8°. The first fixation duration within each AOI during the second phase of the video was extracted. Fixations with durations shorter than 60 ms were discarded. The data were manually inspected on a trial-to-trial basis by a coder (ET) blind to the children’s group membership. We excluded all trials in which the automatically processed and filtered data were not supported by ocular inspection of gaze replays of raw data from the same trial. Gaze shifts during the second phase of the clip were coded as *congruent* when the child shifted gaze from the model to the attended object, and as *incongruent* when the gaze was shifted from the model to the unattended object. Only trials in which the child looked at the model in the beginning of the second phase, when the model was engaging in direct gaze, were included in the analysis. Gaze shifts occurring before the model shifted her gaze were not included. Trials were the child looked at the model but did not fixate any of the objects (i.e. kept fixating the model or looked away) were excluded.

Statistical analyses were performed in SPSS (SPSS Inc., Chicago, IL). Because of the limited sample sizes, non-parametric statistics were used. The analysis included three primary measures. (1) *Accuracy* was calculated as a difference score (DS); the number of incongruent gaze shifts was subtracted from the number of congruent gaze shifts made by each child. (2) A *first fixation duration* *DS* was defined as the duration of the first fixation at the attended object minus the duration of the first fixation at the unattended object. Thus, a DS of zero would indicate no difference in first fixation duration between the two objects. A negative DS indicates a longer first fixation at the unattended object whereas a positive DS indicates a longer first fixation at the attended object. (3) Finally, we assessed the *latency* of gaze shifts from the model to the attended object. Like Bedford et al. (2012), we analyzed only looking time data (first fixation duration) for trials with a congruent first gaze shift. This was also the case with the latency analysis. The reason for excluding trials with incongruent gaze shifts from these analyses is that our focus was to investigate possible differences in performance when the children *did* follow gaze. Five children (ASD = 1; DD = 2; TD = 2) did not shift their gaze from the congruent object to the incongruent object, and were consequently excluded from the analysis of first fixation duration DS.

All tests were two-sided (alpha level = 0.05), with Bonferroni correction for post hoc tests. For the follow-up tests, effect sizes were calculated using Pearson’s *r*.

### Results and Discussion

**Fig. 2:**
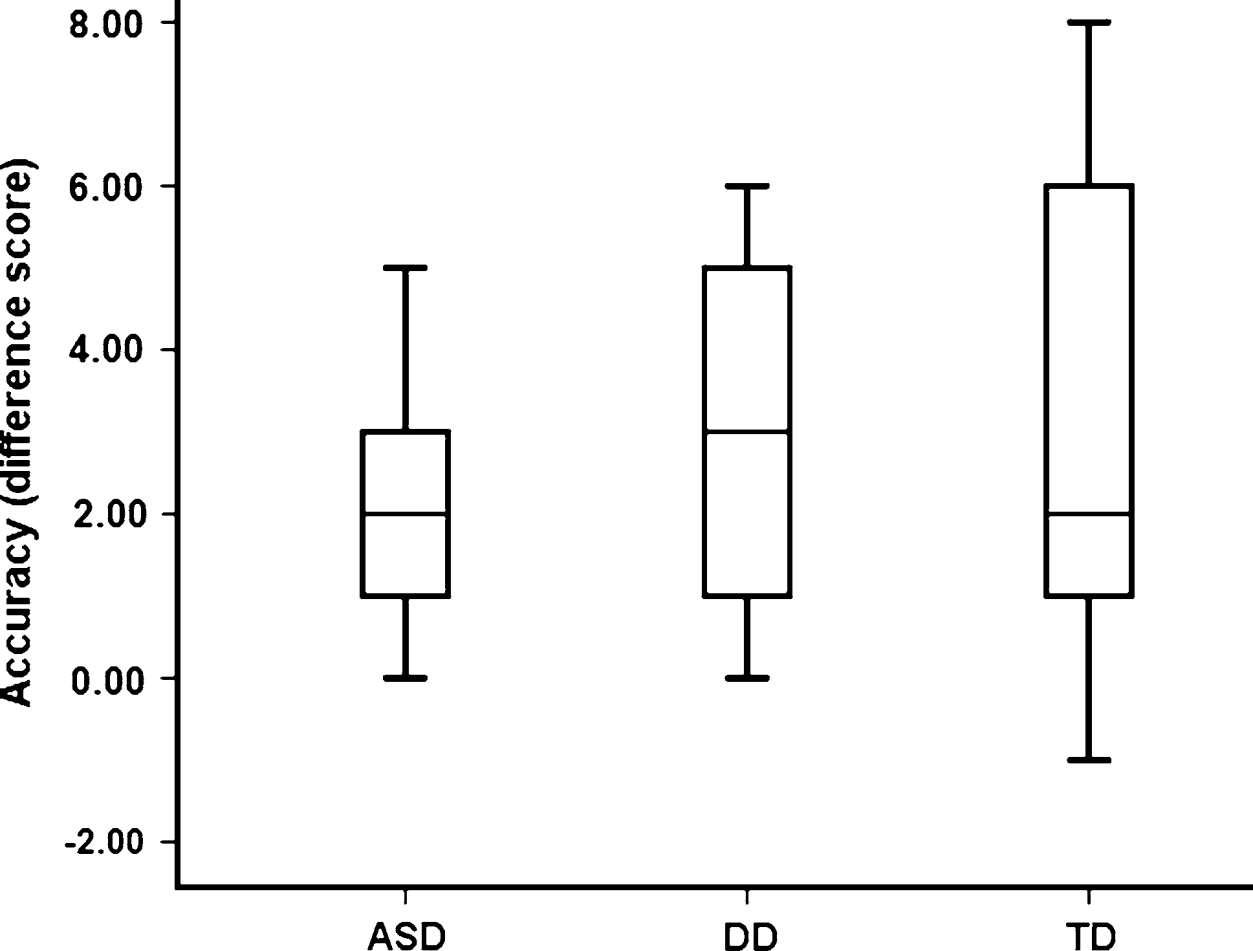
Accuracy (difference score) by group. The difference score was defined as the number of congruent gaze shifts *minus* the number of incongruent gaze shifts. *Box plots* show median, the 75th and 25th quartiles, and the whole data range

An independent samples Kruskal–Wallis test showed that the three groups did not differ from each other in terms of *accuracy*, *χ* ^*2*^(2) = 0.073, *ns* (see Fig. 2). One-sample Wilcoxon signed-rank tests showed that the performance of all three groups differed from chance (ASD, *p* = 0.001; DD, *p* = 0.007; TD, *p* = 0.001). This outcome shows that all groups were able to follow gaze accurately.

In terms of the *first fixation duration difference score*, an independent samples Kruskal–Wallis test showed that the three groups performed differently, *χ* ^*2*^(2) = 9.62, *p* = 0.008. Independent samples Mann–Whitney U tests (Bonferroni-corrected for three comparisons) revealed a weaker first fixation bias for the attended object in the ASD group compared to the DD group (*U* = 8, *p* = 0.012, *r* = 0.660), but no difference between the ASD and TD group (*U* = 37, *ns.*, *r* = *0.413*). The two control groups did not differ from each other (*U* = 25, *ns.* *r* = 0.330).

Next, we analyzed the first fixation duration at the attended and unattended objects separately. For the attended object, an independent samples Kruskal–Wallis test showed that the three groups did not differ from each other, *χ* ^*2*^(2) = 0.975, *ns*. For the unattended object, an independent samples Kruskal–Wallis test revealed that the three groups performed differently, *χ* ^*2*^(2) = 7.191, *p* = 0.027. Independent samples Mann–Whitney U tests (Bonferroni-corrected for three comparisons) showed that the ASD group did not differ from the DD group (*U* = 20.5, *ns.*, *r* = 0.417), but did differ from the TD group (*U* = 31, *p* = 0.033, *r* = 0.511), and that the two control groups did not differ from each other (*U* = 42.5, *ns*., *r* = 0.053).

In terms of *latency*, an independent samples Kruskal–Wallis test showed that the three groups did not differ from each other, *χ* ^*2*^(2) = 0.604, *ns.* For means and SDs for latency and first fixation duration at the attended and unattended objects, see Table 2.

**Table 2:** Means and standard deviations by group for first fixation duration (at attended and unattended objects) and latency measures

Measure	ASD		DD		TD	
	M	SD	M	SD	M	SD
First fixation duration at attended object (s)	0.58	0.31	0.66	0.36	0.51	0.37
First fixation duration at unattended object (s)	0.74	0.44	0.39	0.26	0.33	0.14
Latency (s)	4.00	0.47	4.24	0.80	4.05	0.42

The finding that children with ASD showed no first fixation bias favoring the attended object was in line with our hypothesis. However, the group comparison reached statistical significance only between the children with ASD and the DD group. Although the ASD–TD contrast was in the same direction (and had a medium effect size), it remains a possibility that the results reflected atypical performance in the DD group. Therefore, in Study 2, we included a third reference group consisting of younger typically developing children (TD-toddler group), whose chronological age matched the ASD (and DD) group for mental age. Indeed, it could be argued that the gaze following task is particularly suited for infants and young toddlers and may be too simple or unengaging for older typically developing children.

## Study 2

### Methods

Unless otherwise stated, the methods for Study 2 were identical to those of Study 1. The new participants were 15 toddlers between ages 21 and 23 months (M = 22, SD = 0.5; 5 male, 10 female; final samples after exclusion). The TD-toddlers were recruited from the Uppsala Child and Babylab database. All children were healthy, and none came from a family with a history of ASD. The experimental procedure was identical to that used in Study 1. Because of the young age of the toddlers included in Study 2, we included more trials to obtain reliable measures. Each child viewed 16 trials (no difference in performance was found between the first 8 and second 8 trials). One additional toddler was tested, but excluded because of technical problems.

Because Study 2 was conducted as a follow-up to Study 1 to investigate first fixation durations in a new group of younger children, only first fixation duration data are presented.

### Results and Discussion

**Fig. 3:**
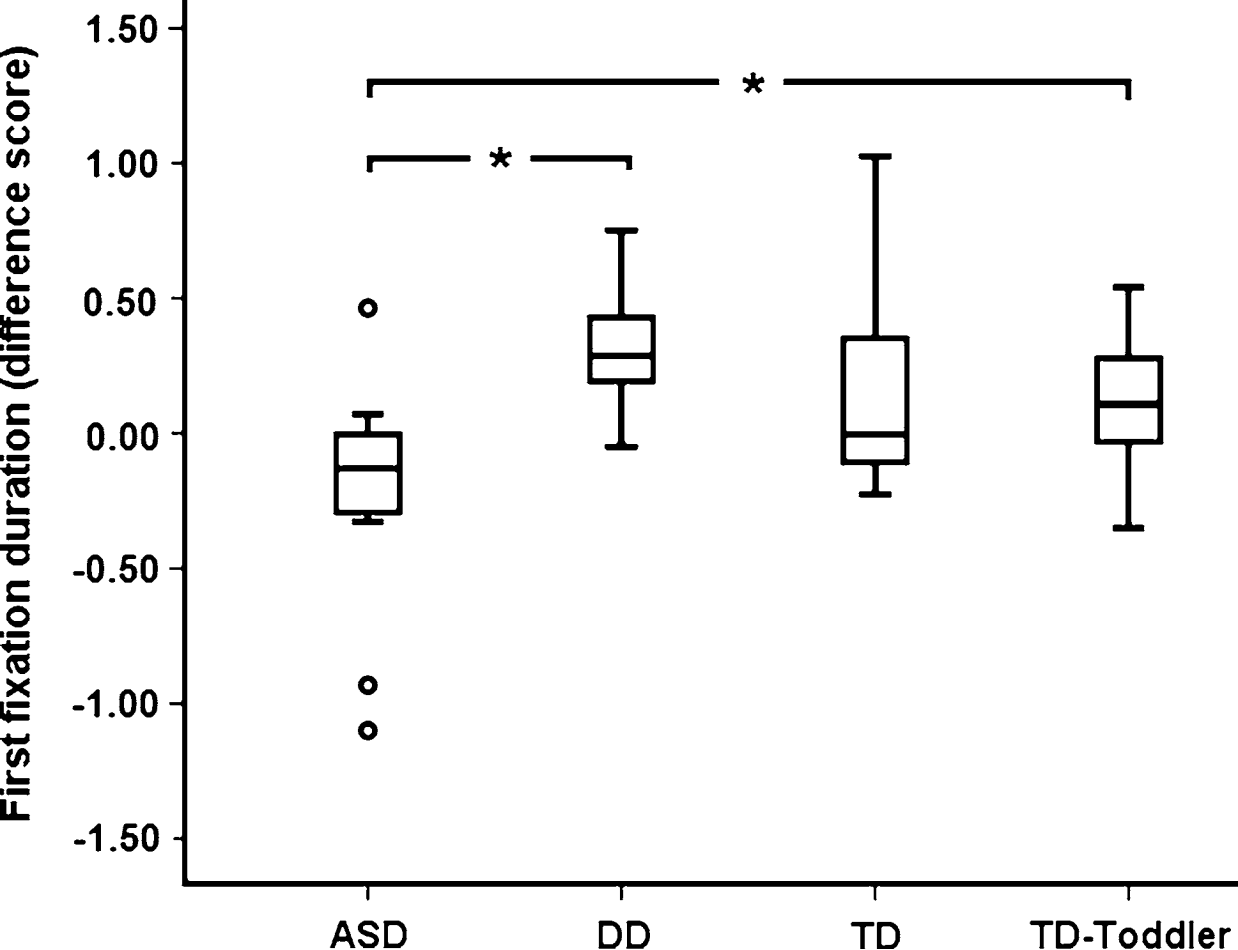
First fixation duration (difference score) by group. The difference score was defined as the duration(s) of the first fixation at the attended object *minus* the duration of the first fixation at the unattended object. *Box plots* show median, the 75th and 25th quartiles, and the whole data range. *p < 0.05

As in Study 1, the dependent measure was the difference in duration between the first fixation at the attended and the first fixation at the unattended object. An independent samples Kruskal–Wallis test revealed that the four groups performed differently in terms of this measure, *χ* ^*2*^(3) = 11.438, *p* = 0.01 (Fig. 3). Most important, a Mann–Whitney U test revealed that the first fixation bias for the attended object was larger in the TD-toddler group than in the ASD group (*U* = 39, *p* = 0.013, *r* = 0.479; mean difference score for the TD-toddler group was 0.12s; SD = 0.24s).

As in Study 1, an independent samples Kruskal–Wallis test revealed that the four groups also performed differently in terms of the duration of the first fixation at the unattended object, *χ* ^*2*^(3) = 9.801, *p* = 0.02. The ASD versus TD-toddler contrast did not reach statistical significance (*U* = 61, *ns.*, *r* = 0.272; Mann–Whitney U test). There was no difference between the girls and boys in the total sample (all groups combined; *U* = 180, *ns*., *r* = 0.250).

The results of Study 2 rule out the possibility that the pattern observed in Study 1 was the result of abnormal performance in the DD group. The results strengthen the conclusion that children with ASD show weaker processing bias for attended objects than do other children. There was no difference between the girls and boys in the total sample (all groups combined; *U* = 180, *ns.*, *r* = 0.250).

## General Discussion

The current study assessed gaze following in low-functioning 3-year-olds with ASD and in typically developing and developmentally delayed children of the same age (Study 1), as well as in younger typically developing toddlers (Study 2). We found no group differences in terms of accuracy of gaze following. Moreover, the ASD, DD, and TD groups made more congruent gaze shifts than expected by chance. This finding is in line with an emerging view that accuracy in gaze following is not impaired in young children with ASD (Bedford et al. 2012). However, in a study of older children with ASD, we recently found reduced gaze following accuracy, possibly suggesting that the motivation to follow other people’s gaze diminishes over early childhood in individuals with ASD (Falck-Ytter et al. 2012).

Of note, the majority of the children in the current ASD sample were also included in another study of perception of biological motion and audiovisual synchrony (Falck-Ytter et al. 2013). In contrast to typical children, a striking lack of preference for these types of information was observed in the autistic group in that study. This result suggests that the ability to follow gaze is preserved in a group of autistic children who are severely impaired in other types of information processing.

Despite pointing to a spared ability to follow gaze accurately in ASD, our fixation duration data suggested that the direction of the adult’s gaze affected initial object processing differently in the four groups. Specifically, the children with ASD showed a weaker first fixation bias for attended objects than both the DD and the TD-toddler groups. There was no significant difference between the ASD and the older TD group, which may reflect that the task was too simple or unengaging for older typically developing children. The group differences are in line with the view that ASD is characterized by a failure to understand the communicative meaning of the joint attention bid (Bedford et al. 2012). That is, despite automatically following gaze to the attended object, children with ASD may fail to subsequently attach a special status to this object. The group difference could also be explained by differences in perception. ASD is associated with feature-oriented perceptual processing, frequently at the expense of configural processing (Falck-Ytter 2008). Thus, compared to children with ASD, non-autistic children may be more influenced by the context during complex scene perception. Specifically, while looking at objects, non-autistic children may be more able to integrate peripheral information about other people’s gaze direction. Whatever the reason, the nature of our dependent measure—the difference in duration between the first fixations at attended and unattended objects—suggests that this altered processing in ASD is detectable on a very short time scale, immediately following the gaze cue.

First fixation duration data were analyzed only for trials in which the children correctly followed the model’s gaze. This design naturally entailed that the attended object systematically was fixated before the unattended object. Pannasch et al. (2008) studied fixation lengths over time in typically developing adults and found a robust pattern of increasingly longer fixation durations during observation of stationary objects (the objects in our study were also stationary). For this reason, we chose not to test the first fixation difference scores against zero because zero is unlikely to be a valid indicator of random performance in this context. It is notable, however, that descriptively (Fig. 1), the ASD group favored the non-attended object, which is the expected direction if no special status is attached to either of the two objects (Pannasch et al. 2008).

Some studies have indicated that young autistic children have problems disengaging their attention from visual stimuli (Elison et al. 2013; Elsabbagh et al. 2013). Such domain general impairments cannot explain why we found a difference in fixation duration between two objects. In addition, altered capacity for disengaging attention should affect the latencies from the model’s face to the attended object, but we found no latency differences. Nevertheless, future studies should attempt to more explicitly include domain general attention measures.

Fixation duration measures can be influenced by data quality, which may vary among child groups (Wass et al. 2013). However, because we contrasted, within each child, the first fixation duration at the attended and unattended object, the differences in fixation durations across groups are unlikely to reflect differences in the quality of the data, assuming that this quality was equal for the attended and unattended objects. To further increase the validity of the study, we manually excluded all trials in which the automatically processed data were not in agreement with the gaze replay of the raw data (‘gaze replay’ refers to showing the stimuli with the gaze trace superimposed). As noted above, contrasting the fixation durations for the attended and unattended object also controls for the possibility of baseline differences in fixation durations during object observation between these child groups.

Sample sizes in the current study were limited, and although we took this into account by analyzing the data using non-parametric statistical tests, independent replication is desirable. Moreover, the gender distribution was not comparable in all groups. However, we found no effect of gender, suggesting that this factor does not explain the difference between the groups.

The ASD group’s SRS scores were significantly higher than the scores of the TD and DD groups, but the scores showed a considerable spread in all groups, with substantial overlap between the two clinical samples. In part, this variability is likely the result of the young age of the participants, as well as the low intellectual level in the DD group. It is also likely that the DD group in fact had elevated levels of ASD symptoms/traits. The results from the experimental task suggest, however, a qualitatively different pattern of social attention in the DD group compared to the ASD group. Although the ASD group showed no bias favoring the attended object, the DD group clearly did.

Taken together, the results of the current study suggest that although autistic children are as accurate as control children in gaze following, the children with ASD show a weaker processing bias for attended objects. Given the important role of social learning in normative child development (Falck-Ytter et al. 2014), this performance—particularly if found during the very first years of life—could be expected to lead children with ASD onto a developmental trajectory that differs fundamentally from non-autistic children. Studies of infants later diagnosed with ASD would be needed to address this hypothesis.
